# Hemoglobin A1C Percentage in Nonhuman Primates: A Useful Tool to Monitor Diabetes before and after Porcine Pancreatic Islet Xenotransplantation

**DOI:** 10.1155/2011/965605

**Published:** 2011-04-17

**Authors:** Marco Marigliano, Anna Casu, Suzanne Bertera, Massimo Trucco, Rita Bottino

**Affiliations:** ^1^Division of Immunogenetics, Department of Pediatrics, Children's Hospital of Pittsburgh of UPMC, 4401 Penn Avenue, Pittsburgh, PA 15224, USA; ^2^Regional Center of Pediatric Endocrinology and Diabetology, “G. Salesi” Hospital, Via Corridoni 11, 60121 Ancona, Italy; ^3^Diabetes Unit, Department of Medicine, Istituto Mediterraneo per i Trapianti e Terapie ad Alta Specializzazione (ISMETT), Via Ernesto Tricomi, 1 90127 Palermo, Italy

## Abstract

Non-human primates (NHPs) are a very valuable experimental model for diabetes research studies including experimental pancreatic islet transplantation. In particular NHPs are the recipients of choice to validate pigs as possible source of pancreatic islets. The aim of this study was to quantify glycated hemoglobin percentage in NHPs and to assess whether changes in values reflect the metabolic trends after diabetes induction and islet transplantation. Sera from 15 NHPs were analyzed. 9 NHPs were rendered diabetic with streptozotocin (STZ), and 3 of them received porcine islet transplants. Hemoglobin A1c (HbA1c) percentage was measured with an assay based on a latex immunoagglutination inhibition methodology. Whereas diabetes and its duration were associated with increasing HbA1c levels, postislet transplantation blood glucose normalization was paralleled by a decrease in the HbA1c percentage. Our data provide evidence that HbA1c is a useful tool to monitor glucose metabolism in NHPs.

## 1. Introduction

Nonhuman primates (NHPs) do not develop autoimmune diabetes. However a permanent type 1 diabetes-like status can be experimentally induced either by total pancreatectomy or by chemical destruction of pancreatic *β*-cells with streptozotocin (STZ) [1, 2]. Both experimental approaches allow for the establishment of chronic hyperglycemia and low endogenous insulin production in NHPs, similarly to what it is found in humans with type1 diabetes. In patients an optimal treatment of diabetes involves control of glycemia by insulin administrations under haemoglobin A1c (HbA1c) monitoring [3]. Daily glucose measurements, even if frequent, do not provide accurate measures of long-term average blood glucose concentrations. The best method to assess long-term glycemic control is the measurement of HbA1c [4]. HbA1c values are important parameters for physicians and quite helpful to adjust the dose of insulin and antidiabetic drugs for a better control of the disease [5]. Evidence supporting the translation of HbA1c into glycemic control and long-term risk assessment of microvascular complications has been provided by the Diabetes Control and Complications Trial (DCCT) [3] and the United Kingdom Prospective Diabetes Study (UPKDS) [6]. The DCCT and the UKPDS are landmark clinical trials that compared the effect of intensive glucose-lowering therapies with conventional blood glucose control on the long-term risks of complications in patients with type 1 (DCCT) [3] and type 2 (UKPDS) [6] diabetes. Both of the trials documented that better glycemic control was associated with improved clinical outcome. 

HbA1c values are strongly correlated with blood glucose levels and with the risk of developing complications. This is the reason why we thought it useful to record HbA1c as a parameter to monitor diabetes in NHPs (particularly in long-term islet graft recipients) as in humans [3]. Even if species differences should be taken into consideration, testing of novel therapeutic approaches in NHPs is one of the best ways to predict possible effects in humans. Clinical signs vary, but there is often a gradual progression of the disease even in NHPs. Traditional tests for the detection of diabetes mellitus include measurement of fasting plasma glucose concentrations, measurement of urine glucose concentrations, oral (OGTT) and IV (IVGTT) glucose tolerance tests, measurement of urine ketone concentrations, and measurement of fasting plasma insulin concentrations [7–11]. Diagnostic criteria are ideally based on the risk of developing long-term microvascular complications [11–14]. While NHPs are the recipients of choice for testing alternative sources of pancreatic islets, such tests present challenges in these animals. These include difficulty in sample collection, necessity for anesthesia during blood drawing with the potential for drug interactions, multiple confounding factors (e.g., activity, duration of food withholding, or diet), stress hyperglycemia attributable to restraint (i.e., catecholamine release suppressing insulin secretion), and lack of established reference ranges [15–17].

Objectives of the study reported here were to identify values for measurement of HbA1c percentage in blood samples obtained from NHPs (*Macaca fascicularis*) to determine whether these percentages varied with respect to glycemic control after diabetes induction and insulin treatment. HbA1c measurements were also carried out after pig islet transplantation in diabetic NHP recipients in an attempt to assess whether this physiologic variable can be considered a suitable test to monitor glucose metabolism and provide a positive feedback after islet transplantation, particularly in long-term survivors. Even if islet xenotransplantation of porcine islets in NHPs restores normal blood glucose levels in diabetic recipients, to date no clear long-term effect has been fully demonstrated. Glycated hemoglobin percentage can offer a reliable means to determine the establishment of euglycemia after xenotransplantation. 

## 2. Materials and Methods

### 2.1. Animals

A total of 15 male Cynomolgus monkeys (i.e., *Macaca fascicularis*, Three Spring Scientific, Perkaise, PA, USA), 2–4 years old and 2.8–4.9 kg (median 3.4 kg), were included in this study; 6 monkeys were nondiabetic, 9 diabetic, and 3 diabetics received islet transplantations. Catheters were placed into the jugular vein and carotid artery. GT-DKO pigs (*α*-1,3-galactosyltransferase double KO pigs) or hCD46 transgenic pigs (Revivicor, Blacksburg, VA, USA) were used as sources of pancreata for islet transplantation. All animal care procedures were in accordance with the institutional Principles of Laboratory Animal Care (National Society for Medical Research) and the Guide for the Care and Use of Laboratory Animals and were approved by the University of Pittsburgh Animal Care and Use Committee. 

### 2.2. Induction of Diabetes

Diabetes was inducted in 9 monkeys with 125–150 mg/kg i.v. of Zanosar Streptozotocin (Sicor Pharmaceutics, Irvine, CA, USA) in a single dose [1]. Diabetes was confirmed by persistent hyperglycemia (>11.1 mmol/L on at least two consecutive readings) and by the need for insulin to prevent ketosis [12]. IVGTTs (intravenous glucose tolerance test) and ASTs (arginine stimulation test) were performed 7–31 days (median 12 days) after induction of diabetes. Diabetic monkeys were treated by continuous i.v. infusion of insulin (Humulin R; Eli Lilly, Indianapolis, IN, USA) to maintain the blood glucose level <11.1 mmol/L and to prevent the development of ketosis. Insulin therapy was stopped 1.5 h before stimulation tests. 

### 2.3. Metabolic Parameters

Blood glucose (mmol/L) was measured in whole blood with a portable glucometer (Freestyle; Abbott Laboratories, Abbott Park, IL, USA). Serum levels of primate C-peptide (nmol/L) were measured by radioimmunoassay (Linco Research, St Charles, MO, USA) using species-specific antibodies. Aprotinin 0.05 kIU/l (Trasylol; Bayer Pharmaceuticals, West Haven, CT, USA) was added to the serum as a stabilizing agent. To document the metabolic status of each monkey before induction of diabetes, after induction of diabetes, and after islet transplantation until euthanasia, we recorded mean blood glucose (mmol/L), the prevalence of blood glucose readings >11.1 mmol/L (%), the mean exogenous insulin requirement (IU kg^−1^ day^−1^), and mean porcine (graft) C-peptide levels [18].

Blood samples for HbA1c testing were collected fasting every 2–4 weeks before and after induction of diabetes. Immediately after collection of a sample, blood was transferred into a tube containing EDTA, which was immediately used for measurement of HbA1c percentages. For the measurement of specific HbA1c, an inhibition of latex agglutination assay is used (HbA1c-specific mouse monoclonal antibody adsorbent onto latex particles, DCA Vantage Analyzer, Siemens Healthcare Diagnostics, Deerfield, IL, USA). An agglutinator (synthetic polymer containing multiple copies of the immunoreactive portion of HbA1c) causes agglutination of latex coated with HbA1c specific mouse monoclonal antibody. This agglutination reaction causes increased scattering of light, which is measured as an increased absorbance at 531 nm. HbA1c in whole blood specimens competes for the limited number of antibody-latex binding sites causing an inhibition of agglutination and decreased scattering of light. The decreased scattering is measured as a decrease absorbance at 531 nm. The HbA1c concentration is then quantified using a calibration curve of absorbance versus HbA1c concentration. The percent HbA1c in the sample is then calculated as follows: %HbA1c = [HbA1c]/[Total Hemoglobin] × 100. 

### 2.4. Porcine Islet Isolation and Transplantation into Monkeys

After pancreatectomy in the anesthetized donor pig, islet isolation was carried out according to a modification of the method described for human islets, optimized for pigs [19] that involved low enzyme concentration, low digestion temperature, and minimal mechanical digestion. Intraportal injection of islets (an average of 40,000–100,000 islet equivalents/kg body weight) was carried out under general anesthesia of recipients. Continuous insulin infusion was restored if blood glucose was consistently >11.1 mmol/L. After induction with antithymocyte globulin, immunosuppression was maintained with humanized anti-CD154 monoclonal antibody (ABI 793, Novartis Pharma, Basel, Switzerland) and mycophenolate mofetil (Roche, Nutley, NJ, USA) [18].

Anticoagulation/antiaggregation/anti-inflammatory treatment was achieved with heparin or dextran sulfate, prostacyclin (GlaxoSmithKline, Research Triangle Park, NC, USA) and aspirin [18]; islet graft function was monitored by measuring porcine C-peptide. 

### 2.5. Statistical Analysis

A commercially available technical computing program was used for graphic analyses (GraphPad Prism 4 for Macintosh GraphPad Software, La Jolla, CA, USA). Suggested criteria for diabetic classification of subjects were derived from other studies [7, 15] that involved the use of NHPs. A commercially available statistical program was used for statistical analyses (GraphPad Prism 4 for Macintosh, GraphPad Software). Experimental data are presented as means ± SE. Human data obtained from the literature are presented as the range of values or mean of the published data [20, 21]. 

## 3. Results

### 3.1. Comparison of Metabolic Parameters between Nondiabetic NHPs and Humans

Fasting HbA1c values measured in nondiabetic monkeys are presented in Table 1. The data for healthy monkeys were compared with the human data from the literature to better characterize similarities and differences, even though the comparisons are limited by the difference in the testing conditions. For a better characterization of differences between monkeys and humans Table 1 also reports fasting blood glucose and C-peptide levels comparisons, based on previous reports [18]. Blood glucose in fasting nondiabetic monkeys ranged from 3.1 to 4.9 mmol/l and was significantly lower than the corresponding values in humans [21] (3.9–5.6 mmol/l; *P* < .05). Human C-peptide [20] values were consistently lower than monkey C-peptide levels (0.47  ±  2.93 nmol/l in monkeys versus 0.17–0.66 nmol/l in humans, *P* < .05). Furthermore, HbA1c values for nondiabetic healthy monkeys were lower than those in humans [22], with statistically significant difference (4.4 ± 0.1% in monkeys versus 4.99 ± 0.1% in humans, *P* < .05). 

### 3.2. Comparison of Metabolic Parameters between Nondiabetic and Diabetic NHPs

**Figure 1:**
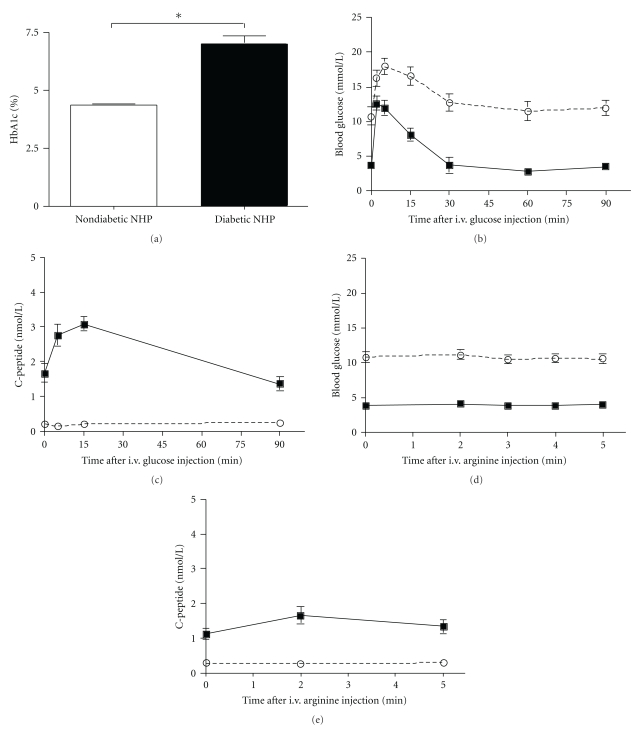
Comparison of metabolic parameters between nondiabetic and diabetic monkeys. (a) HbA1c comparison between non diabetic NHPs and post-STZ diabetic NHPs (*P < .05). Mean ± SE of blood glucose levels (b, d) and primate C-peptide levels (c, e) during IVGTT (b, c) and AST (d, e) in nondiabetic (solid lines) and diabetic (broken lines) monkeys. The primate C-peptide response to physiological stimuli was completely abolished in diabetic monkeys. This was associated with a prolonged increase in the blood glucose level.

The HbA1c of nondiabetic monkeys was compared to that of monkeys that were streptozotocin (STZ) induced, hyperglycemic, and insulin independent. The increase in HbA1c levels following diabetes induction confirms also in NHPs the notion that chronically high blood glucose affects glycation of hemoglobin (Figure 1(a), *P* < .05). The diabetic status was characterized using standard metabolic tests. IVGTT and AST were performed in diabetic NHPs and compared to nondiabetic controls. As expected, during the IVGTT the peak of glucose concentration in diabetic monkeys was significantly higher (*P* < .05, 2 min after glucose i.v. infusion) than in nondiabetic monkeys (Figure 1(b)). Thereafter, the glucose levels decreased at a slower rate in diabetic than nondiabetic animals, as shown by the lower *K*_*G*_ (mean 1.44 ± 0.2 mmol^−1^ min^−1^, *P* < .001). The C-peptide increase, seen in nondiabetic monkeys, was absent in diabetic monkeys (Figure 1(c)).

During the AST in nondiabetic monkeys (Figures 1(d) and 1(e)), blood glucose remained stable while C-peptide values rose at 2 min and then returned at prestimulus value at 5 minutes. The ACR_Arg_ ranged from 0.04 to 1.09 nmol/L. Published data show that human ACR_Arg_ is similar to that in monkeys; however, the absolute basal and stimulated values are lower in humans than in monkeys (22–23). In diabetic monkeys during the AST, blood glucose remained stable at approximately 10.8 mmol/L, and C-peptide showed no response (ARC_Arg_−0.40–0.31 nmol/L). In summary, after STZ treatment, blood glucose levels in monkeys increased above 10 mmol/L, and fasting levels of endogenous C-peptide declined to values corresponding to 12–18% of the C-peptide levels before diabetes induction. Insulin was needed to maintain blood glucose <11.0 mmol/L and to prevent ketosis. Any residual endogenous C-peptide did not respond to physiological stimuli, as shown by the results of the dynamic tests and by the absence of a correlation between endogenous C-peptide and blood glucose levels. 

### 3.3. Comparison of Metabolic Parameters in Diabetic NHPs before and after Porcine Islet Transplantation

**Figure 2:**
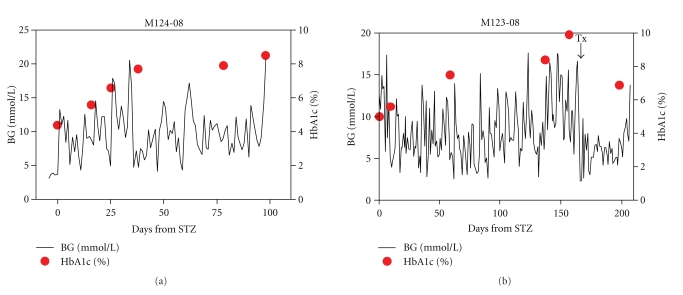
Blood glucose levels (solid lines) and HbA1c (%) (red dots) in STZ diabetic NHP (a) and STZ diabetic with porcine islet graft NHP (b). In (a) the HbA1c values are increasing overtime despite exogenous insulin treatment; in (b) the HbA1c value drops after porcine islet transplant.

Figure 2 compares the blood glucose profile and HbA1c levels in two NHPs, during their followup after STZ and (in one case) islet transplantation.  Figure 2(a) shows the blood glucose levels trend after STZ of monkey M124–08. Despite IV insulin treatment blood glucose level tended to increase, and coherently HbA1c values (red dots) also tended to increase (from a basal pre-STZ value of 4.3% to 8.2% at the end of followup).  Figure 2(b) shows a second monkey (M123-08) blood glucose profile and HbA1c values prior to and after islet transplantation. Whereas blood glucose control and HbA1c increased after STZ, as also shown in monkey M124-08, a substantial decrease in blood glucose levels and a drop in the HbA1c level from 9.9% to 6.5. was recorded as effect of the islet transplantation. Better physiological glycemic control was achieved by the islet transplant and was reflected in the improved HbA1c values, testifying to a better management of the disease.

**Figure 3:**
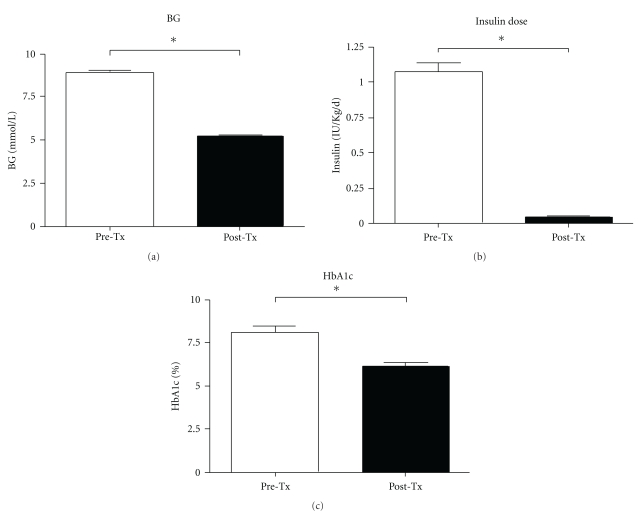
Comparison between blood glucose levels (mmol/L) (a), insulin dose (IU kg^−1 ^day^−1^) (b), and HbA1c (%) (c) in NHPs before and after porcine islet transplantation. There is a statistically significant difference (*P < .0001).

In this experiment a total of three monkeys were transplanted with porcine islets in the liver via the portal vein; following the transplant, all of them experienced improved metabolic control of glucose. Improvement was defined by a statistically significant drop in between blood glucose levels (8.87  ±   0.19 mmol/L versus 5.28  ±   0.06 mmol/L, *P* < .0001) and insulin dose (1.07 ± 0.06 IU Kg^−1^ day^−1^ versus 0.04 ± 0.01 IU Kg^−1^ day^−1^, *P* < .0001). Paralleling these parameters, a statistically significant decrease in HbA1c percentage (8.11 ± 0.36 versus 6.16 ± 0.21, *P* < .0001) was found before and after islet transplantation (Figures 3(a), 3(b), 3(c)). 

**Figure 4:**
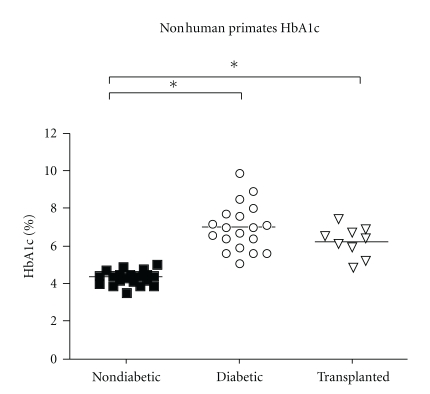
Comparison between HbA1c % levels in nondiabetic, diabetic, and porcine islet transplanted NHPs. A statistically significant difference is shown between nondiabetic and diabetic animals and nondiabetic and islet transplanted animals (*P < .001).

Figure 4 shows a summary of the-HbA1c values comparing nondiabetic, diabetic, and islet-transplanted NHPs. There is a statistically significant difference in HbA1c levels between nondiabetic and diabetic NHPs and between nondiabetic and islet-transplanted NHPs (*P* < .0001), showing a trend of improvement of HbA1c values that parallels the improvement of blood glucose levels, as expected. However, caution is needed in interpreting these results because of the short time between samples. 

## 4. Discussion

Glycated hemoglobin (Hb) is produced when a carbohydrate, such as glucose, binds to an Hb molecule, such as HbA_0_, and undergoes intermolecular transformation to form a stable glycated ketoamine product [23].

HbA1c is formed via a posttranslational nonenzymatic attachment of glucose to hemoglobin at a rate dependent on the ambient blood glucose during the lifespan of the red blood cells (approximately 120 days in humans). Glycated Hb percentage represents therefore the integrated value for average blood glucose concentrations in the preceding 6 to 8 weeks [10]. A number of different glycated Hb forms have been identified, reflecting various attached sugar residues. These forms include HbA1a (fructose-1,6-diphosphate, or glucose-6-phosphate), HbA1b (ketamine-linked pyruvic acid), and HbA1c (glucose) [4]. This last form is the most commonly tested, constituting approximately 80% of hemoglobin A1 (HbA1). This hemoglobin is a derivate of adult hemoglobin (HbA), with monosaccharide (fructose or glucose) attachments. In strict chemical terms, the molecular structure of HbA1c is N-(1-deoxy)-fructosyl-hemoglobin or N-(1-deoxyfroctose-1-yl) hemoglobin beta chain. Methods routinely used for the measurement of glycated Hb percentage separate the molecule on the basis of its charge, structure, or antigenic properties [10]. The most popular methods rely on the increased negative charge found in the glycated Hb molecule to distinguish it from its nonglycated form. These assays include electrophoresis and ion-exchange chromatography as by high-performance liquid chromatography (HPLC). Alternatively, boronate affinity chromatography separates glycated Hb on the basis of its structure rather than its charge. In this assay, separation is the result of carbohydrate moieties on the glycated Hb molecule binding by condensation to the affinity reagent, di-hydroxyboronate. This method is specific for all glycated Hbs, irrespective of molecular charge or the site of glycation on the Hb molecule. In addition, it is also able to detect the glycated portion of Hb in patients with Hb variants such as Hb S, C, or F. Thus, the term total glycated Hb has been used to describe this type of assay. Finally, immunoassays, such as the method we have utilized in this study, have been developed for the measurement of HbA1c. Monoclonal or polyclonal antibodies generated against the 6- to 8-amino acid peptide of the glycated amino terminus of the *β*-globin chain are here utilized. Advantages to measure HbA1c percentage with this technology involve the simplicity of the approach; the need for one small (only few microliters) blood sample; the fact that the assay is relatively unaffected by recent food intake, activity, illness, or stress; it still reflects glycemic control during the preceding few weeks [10] allowing measurement of HbA1c even when pathological hemoglobin variants or NHP hemoglobins are involved. Unfortunately, there are also a lot of different variables associated with measurement of glycated Hb percentage that could potentially modify those values; for example, processes that decrease the mean lifespan of RBCs will reduce the availability of Hb for glycation and, therefore, the percentage of glycated Hb, irrespective of glucose concentrations. Similarly, conditions that increase the lifespan of RBCs (e.g., iron deficiency) will increase the amount of glycated Hb. Appropriate validation is therefore required for experimental animal models such as diabetic NHP recipients of pig islet xenotransplantations. Reliable measurement of glycated Hb percentages would be then useful in determining the efficacy of therapeutic treatments.

Glycated Hb percentages have been measured, only to a limited degree, in non diabetic, borderline-diabetic, and confirmed-diabetic NHPs [17, 24–26]. These studies have involved a relatively low number of species and various methods. Percentages of HbA1 and HbA1c in nondiabetic baboons (*Papio anubis*), measured by use of cation-exchange chromatography, are approximately half of those found in humans [24]. It has been suggested that this may be explained on the basis of differences in the life-span of RBCs in humans (100 to 120 days) compared to baboons (30 to 60 days). Variations may also be related to differences in permeability of RBCs to glucose [17]. At least 2 studies [25, 26] have documented an increase in HbA1c percentages in borderline-diabetic or confirmed-diabetic NHPs. In one group of Celebes crested macaques (*Macaca nigra*), the HbA1c value increased from 2.6% in nondiabetic macaques to 7.9% in diabetic macaques, as measured by use of electrophoresis [25]. In addition macaques without overt hyperglycemia, but with impaired glucose clearance, impaired insulin secretion, and increased postprandial glucose concentrations, had a significant increase in HbA1c content to 3.5%. Reference ranges for glycated Hb percentages have not been determined in many NHPs, but values that increase by ≥10% were generally considered to be abnormal [7]. Although the number of animals that were evaluated in our study is small, also reflecting the complexity of the experimental model, we had the opportunity to use the same technology to measure variations in HbA1c values within the same animals under different clinical metabolic conditions: prior to diabetes induction, as insulin-dependent diabetics, and after islet transplantation.

The levels of HbA1c obtained reflected different glucose metabolic conditions such as diabetes, and improved metabolic control following islet transplantation. These values coherently represent the expected trends relative to the experimental metabolic conditions and further validate the technical approach proposed. By using this simple method in broader scale studies and employing larger numbers of animals, a better correlation between HbA1c levels and blood glucose levels in Cynomolgus monkeys can be achieved. 

## 5. Conclusions

In this study we presented the HbA1c values of nondiabetic and diabetic NHPs (*Macaca Fascicularis*), analyzed with an inhibition of latex agglutination assay (HbA1c-specific mouse monoclonal antibody adsorbent onto latex particles). These values are lower than the ones we find in humans without diabetes and very similar to those in diabetic patients under insulin treatment, respectively. Total HbA1c percentage can be measured with a user friendly assay and provides useful information for control of diabetes mellitus in NPHs, as is done in human patients, and it could be used during the standard management of glucose metabolism in those animals. Our data provide evidence that HbA1c can be used to monitor diabetes in streptozotocin-diabetic monkeys and to receive feedbacks on the functionality of xenogenic islet transplantion, particularly useful during long-term followups. 

## Figures and Tables

**Table 1:** Fasting blood glucose, C-peptide and HbA1c in monkeys and humans.

	Cynomolgus monkeys	Humans
Blood Glucose (mmol/l)	3.1–4.9 (3.7 ± 0.1,*n* = 31)^a^	3.9–5.6 [21]
C-peptide (nmol/l)	0.47–2.93 (1.40 ± 0.17,*n* = 18)^a^	0.17–0.66 [20]
HbA1c (%)	3.5–5.0 (4.4 ± 0.1,*n* = 23)^a^	4.46–5.52 [22]

Data are ranges, with means ± SE and number of measurements in parameters. Human data were obtained from the literature and were measured in venous plasma [20–22]. Blood glucose levels are significantly lower in monkeys than in humans. C-peptide levels are significantly higher in monkeys. There is statistically difference regarding HbA1c levels. (Monkeys versus humans, ^a^ *P* < .05).
